# Titanium-based nanocomposite materials for arsenic removal from water: A review

**DOI:** 10.1016/j.heliyon.2019.e01577

**Published:** 2019-05-15

**Authors:** Sobia Ashraf, Asima Siddiqa, Shabnam Shahida, Sara Qaisar

**Affiliations:** a Department of Chemistry, University of Poonch Rawalakot, Azad Kashmir, Pakistan; b NanoScience and Technology Department, National Centre for Physics, Islamabad, Pakistan

**Keywords:** Environmental science, Engineering, Materials science, Chemistry

## Abstract

Arsenic is highly carcinogenic element and less concentration of this chemical element makes natural water unsafe for human consumption. Versatile techniques including adsorption method have been established to remove the arsenic from water. However, adsorption is found to be one of effective method for the remediation of arsenic from contaminated water. Different types of natural adsorbents i.e. clays, waste materials, carbon based material have been studied widely for the adsorption of arsenic. Recently, nanotechnology is considered to be one of the best technology for waste water treatment. Therefore researchers have synthesized several types of nanoadsorbents and investigated them for the removal of various pollutants including arsenic from water. Now days, attention is paid on development of nanocomposite materials which are proven as competent arsenic adsorbent candidate as compared to other adsorbents due to dominant structural and surface features. Various metal/metal oxide based nanocomposites have been developed and studied for arsenic removal from aqueous media. It has been reported that TiO_2_ based nanocomposite exhibit stong affinity for both inorganic form of arsenic. Therefore, in this review numerous metal or metal oxide based titania nanocomposites i.e. TiO_2_-αFe_2_O_3_, NHITO, Ce-Ti oxide, Zr-TiO_2,_ RGO-MFT etc. have been discussed in details for the water treatment containing arsenic. This review also presents an overview of low cost adsorbents, titania based nanoadsorbent and hybrid titania nanostructures for the removal of arsenic. In this review paper the particle size, surface area and adsorption efficiency of these titania based materials at different pH are also been presented in tabulated form. It provides the opportunity to choose best titania based nanocomposites for the treatment of arsenic polluted water.

## 1 Introduction

Arsenic is the 20^th^ toxic element on earth crust, it is 14^th^ and 12^th^ abundant component in sea water and human body, respectively [1, 2]. Both organic and inorganic forms of arsenic exists in the environment but inorganic forms (As (III) and As (V)) are present in high concentration than organic forms i.e. monomethylarsonic acid (MMA) and dimethylarsonoic acid (DMA) [3, 4, 5]. Inorganic arsenic usually exists in -3, 0, +3 and +5 oxidation states. The toxicity of various arsenic species increases in this order such as arsenite > arsenate > MMA > DMA. Arsenite is more lethal to human health than arsenate because it readily adsorbed in the body and has high affinity for sulfurhydral groups and inhibits the action of enzyme [6, 7]. The existence of arsenic trioxide (As_2_O_3_) and arsenic pentaoxide (As_2_O_5_) in water depends upon pH of aqueous media. It is found that arsenic trioxide (As_2_O_3_) dominant under neutral pH and anaerobic conditions, however, As (V) dominates in aerobic conditions under basic pH [6]. In addition to natural sources (such as rain and wind), arsenic penetrates into water by the burning of fossil fuels, use of pesticides, herbicides, dried crops and feed of livestock [8, 9, 10]. Drinking of As contaminated water has a tremendous impact on human health and cause innumerable serious illness like cancer, melanosis, hyperkeratosis, restrictive lung disease, gangrene, hypertension, type 2 diabetes mellitus and peripheral vascular disease. It has become a big issue for the whole world as concentration of arsenic in water is increasing day by day [11, 12, 13, 14, 15, 16].

Several techniques have been employed to remove arsenic from drinking water and these include, membrane separation techniques (mixed matrix reverse osmosis membranes i.e. formed by incorporation of inorganic materials like TiO_2_ into thin film membranes) [17], coagulation-precipitation, adsorption, electrochemical methods (electrocoagulation & electrodialysis), photocatalysis (heterogeneous catalytic method) and ion-exchange [18, 20, 21, 22, 23, 98, 99]. Advantages and disadvantages of all these techniques are compared in Table 1.

**Table 1:** Comparison of advantages and disadvantages of different technologies.

Methods	Advantages	Disadvantages	References
Air oxidation	Easy and low cost, oxidizes other organic and inorganic constituents in water	Slow process and increases the rate of oxidation, eliminates arsenic (v) mostly.	[19]
Chemical oxidation	Natural process, operates rapidly, cause oxidation of other impurities and minimum waste production.	Depend highly on pH and requires oxidation step.	
Adsorption (activated alumina, iron oxides, titania etc.	Simple and easily controlled. No other reagents and additional steps required. Adsorbent can be regenerated.		[25]
Nano-filtration	High removal capacity. Well- defined process.	Economically not effective process, high water rejection.	[17]
Reverse osmosis	Toxic waste is not produced. High efficiency for the removal of arsenic.	Difficult to operate.	
Electrodialysis	Can also be used for the removal of other pollutants.	Production of toxic waste water.	[23]

### 1.1 Adsorption method for the removal of arsenic

Adsorption is one of the best technique for the removal of arsenic from drinking water because it is cost-effective [25], convenient [26], efficient [27] with minimal waste production [28], however, other techniques are found to be expensive, difficult to operate, cannot removed maximum amount of metal ions and produce toxic waste [24, 89].

**Fig. 1:**
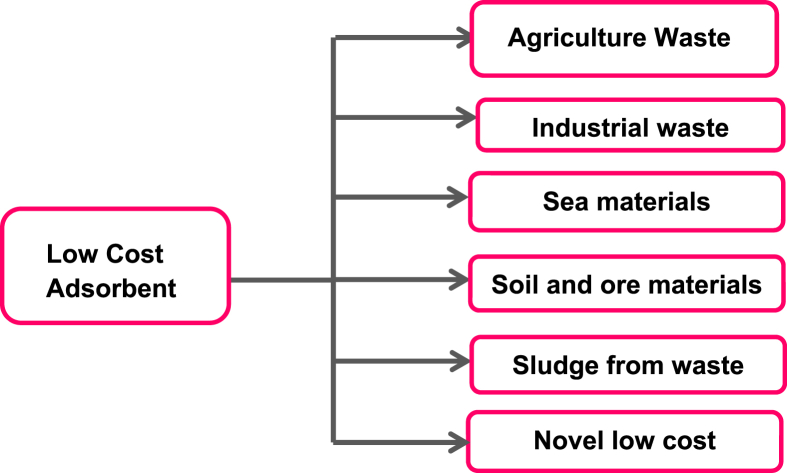
Low cost adsorbent for the removal of arsenic.

Adsorption process depends on different parameters such as adsorbent concentration, temperature, contact time, pH and electrostatic force between the adsorbate and adsorbent [29, 30, 31]. Adsorbent surface area is a prominent and significant factor in the surface processes. Organic and inorganic forms of arsenic react relatively well with the adsorbent in different ways such as organic forms of As adsorbs due to hydrophobic and weak intermolecular forces while inorganic forms of As react with the functional group of adsorbents [32]. Many classes of nano-adsorbents, nano-catalysts and biologically active nanoparticles are being used for the adsorption of arsenic from contaminated water [33]. Activated carbons can also obtained from waste of tires and proved as efficient adsorbent for the removal of pollutants including arsenic, chromium (III), but it cannot applied at industrial level due to its high cost. Other low cost dsorbents used for the removal of arsenic include dry plants, red mud, fly ash, zeolites, blast furnace slags, hydrotalcites, hydroxides, rice husk [34, 35, 84, 88, 93] and natural clays but these adsorbents has disadvantage that they cannot be regenerated [18, 36, 37, 95]. Fig. 1 presents the low cost adsorbents and its types.

The nanoparticles possess special properties such as high surface area, high reactivity, high specificity and regeneration capability due to which researchers have given more attention on the use of nanomaterial for environmental remediation [38]. Recently, widely used nanoparticles include carbon based materials (CNTs/MWCNTs, and graphene etc.), metals (Ce, Zr and Ti etc.) and metals oxides (CuO, Fe_2_O_3_/Fe_3_O_4_, TiO_2_, ZnO and CaO etc.) based nanomaterials for the treatment of arsenic contaminated water [39, 40, 84]. Researchers found that different metal oxide nano-adsorbents have high adsorption capacity for the both inorganic forms of arsenic i.e. As (V) and As (III). The drawback associated with these nanoparticles is agglomeration due to low energy barrier, however this problem can be overcome by impregnation method and surface coating or doping of nanoparticles with the various types of surfactants (anionic, cationic and nonionic), polymers (polyethylene glycol etc.), polyelectrolytes and different metal or metal oxide nanoparticles [28, 41, 42, 43, 44]. Literature study showed that titanium dioxide (Ti$O_{2}$) has ability to remove arsenic and exhibits promising results than other metal oxides due to its low toxicity, chemical and physical stability, easy preparation, low-cost, environment friendly and high affinity for both inorganic forms of As (arsenite and arsenate) [3, 18, 45, 46, 47]. However, it has certain limitations such as surface area of titania decreases due to agglomeration which can be overcomed by forming its nanocomposites or coating with other transition metals or metal oxides. Titania based nanocomposites have large surface area and efficient adsorption capability than individual TiO_2_ nanoparticle [48, 49, 50]. The structure of nanoparticles has a significant impact on the adsorption, crystalline form of the Ti$O_{2}$has more adsorption capability than amorphous form [51, 52, 53]. Extended X-ray absorption fine structure (EXAFS), fourier transform infrared spectroscopy (FT-IR), electrophoretic mobility and charge distribution multi complexation (CD-MUSIC) model are employed for the description of mechanism of adsorption of As (III) and As (V) on nano-crystalline titanium dioxide [54]. It was investigated by EXAFS that titania formed monodentate and bidentate complexes with the both prominent inorganic (i.e. arsenite & arsenate) and organic forms (MMA & DMA) of arsenic. According to CD-MUSIC, a singly coordinated surface group (TiO$H^{1/3}$ ) was responsible for the adsorption of arsenic [55].

The focus of this review is on titania and titania based metal or metal oxide nanocomposites for the removal of arsenic from drinking water. In this study, various forms of TiO_2_ such as amorphous TiO_2_, nanocrystalline TiO_2_, granulated TiO_2_, and Ti${\text{O}}_{2}$-based metal oxide nanocomposites are discussed in detail as shown in Tables 2 and 3.

**Table 2:** Comparison of hybrid titania nanostructures for the adsorption of arsenic.

Materials	Particle size (nm)	Surface area (m2/g)	pH	Adsorption capacity (mg/g)	Forms of Arsenic	References
Hydrous TiO_2_	3–8	312	7.0 9.0	83 96	As(III)	[56]
Hydrous TiO_2_	10.8 mm	280	4.0 4.0	33.4 31.8	As(V) As(III)	[57]
Granular TiO_2_	0.15–0.6 mm	250.7	7.0	41.4 32.4	As(V) As(III)	[58]
CHTO	0.14–0.29 mm	163.6	7.0	72–75 g/kg	As(III)	[57]
TICB	…	0.56	7.0	2050 μg/g 2099 μg/g	As(V) As(III)	[59]
TICB under UV-light	…	3.06	7.0	2988 μg/g 3536 μg/g	As(V) As(III)	[60]
TNTs	2–6	312	7.0 3.0	60 208	As(III) As(V)	[62]
TiO_2_-CNTs	2.7	196	7.0	1.8 1.3	As (III) As (V)	[63]
TiO_2_/MMT Dark	1.83–4	176.4	7.0	4.58 4.86	As(III As(V)	[66]
TiO_2_/MMT UV	…	…	…	5.19 5.16	As(III) As(V)	…

Abbreviations: CHTO, crystalline hydrous titanium oxide; TICB, titania impregnated chitosan bead; TNTs, titania nanotubes CNTs, carbon nanotubes; MMT, monotomrolonite.

**Table 3:** Evaluation of metal/metal oxide based titania nanocomposites for the removal of arsenic.

Materials	Particle Size (nm)	Surface Area (m^2^/g)	pH	Adsorption capacity (mg/g)	Forms of arsenic	References
m- TiO_2_-αFe_2_O_3_	26.7	95	3.0 7.0	80% 99%	As(III) As(V)	[67]
TiO_2_-Fe_2_O_3_ bi-composite	20 μm	133.5	5.0 7.0 9.0	12.4 7.79 6.48	As(V)	[68]
Fe-TNTs	8–11	162.8	2.5	80.67 36.41	As(V)	[69]
NHITO	7.0–11	77.8	7.0 7.0	85 14.3	As(III) As(V)	[70]
RGO-MFT	20–45	275.23	6.0 7.0	77.6 99.5	As(V) As (III)	[71]
FTSZ	61 nm	189.2	7.0		As (III)	[72]
Ce-Ti oxide	100–200 nm	38.2–68.8	6.5 6.5	6.8 7.5	As (III) As (V)	[78]
Zr-Ti oxide	……	114	9.0 3.0	28.6	As(III) As (V)	[80]
Ti-BYC	10–30 nm	82	7.0	348.5	As(V)	[82]

Abbreviations: TNTs, titania nanotubes; NHITO; Iron (III) titanium (IV) mixed binary oxide; RGO-MFT, reduced graphene oxide/magnetite iron-titania ternary; FTSZ, iron, titania/silica modified with zinc.

## 2 Main text

### 2.1 Hybrid titania nanostructures for the adsorption of arsenic

Titania has wide range of applications in waste water treatment and proved to be an effective adsorbent. Various nanostructures of TiO_2_ has been developed with different compositions such as homogeneous structures (hydrous titania, crystalline and granular) or heterogeneous structures (Ce-Ti, Zr-Ti and Fe-titania etc.) to enhance the adsorption properties of it. It has been reported that several titania nanocomposites (Mn_2_O_3_/TiO_2_, CuO–TiO_2_, WO_3_–TiO_2_, ZnO/TiO_2_ and TiO_2_/MgO) also employed as a photocatalyst for degradation of pollutants (either organic or inorganic) and various types of textile dyes [52, 91, 94, 97].

Zhengchao *et al.,* synthesized Ti$O_{2}$.xH_2_O through single step hydrolysis process and then carried out adsorption experiment to study the effect of pH on the adsorption efficiency of hydrous titania for arsenite. The surface area of this particle was 312$\;m^{2}/$g, diameter(∼) 3–8nm and had a very porous structure. It showed the maximum adsorption capacity for As (III) when pH was increased from 8 to 9 [56]. Biswaranjan Manna *et al.* prepared crystalline hydrous titanium dioxide by desalination of titanium tetrachloride and noticed its adsorption capacity for arsenic (III). Experiment was also performed to evaluate the effect of pH on the adsorption of As (III), which showed that adsorption increased until pH 6, remained constant up to pH 9. This adsorbent was also used to remove manganese along with arsenic from water [57]. Bang *et al.*, investigated the ability of adsorption of granular Ti$O_{2}$ for As (III) and As (V) and experiments (batch adsorption and field filtration tests) were conducted to interpret the effect of other anions and pH on the adsorption capacity of granular titania. The results indicated that adsorption capability of granular titania decreased for As (V) from 99 % to 70% when the pH was increased from 7.3 to 10.3. Moreover, the presence of anions in water also reduced the removal efficiency of arsenic on granular titania [58].

#### 2.1.1 Chitosan based titania adsorbent

Chitosan is a biopolymer that can be transformed into beads and films by simple process; however, it is also used for the removal of metals cations from water [35]. Although, adsorption capacity of chitosan for arsenic was not so high, it has some advantages such as biodegradable, non–toxic and easily available from shellfish industries. Adsorptive properties of chitosan can be enhanced by forming composites with Fe_3_O_4_/Fe_2_O_3_, graphene, polymer based material, clay and cellulose. Chitosan based composites are also employed for the removal of dyes and heavy metals from water [18].

**Fig. 2:**
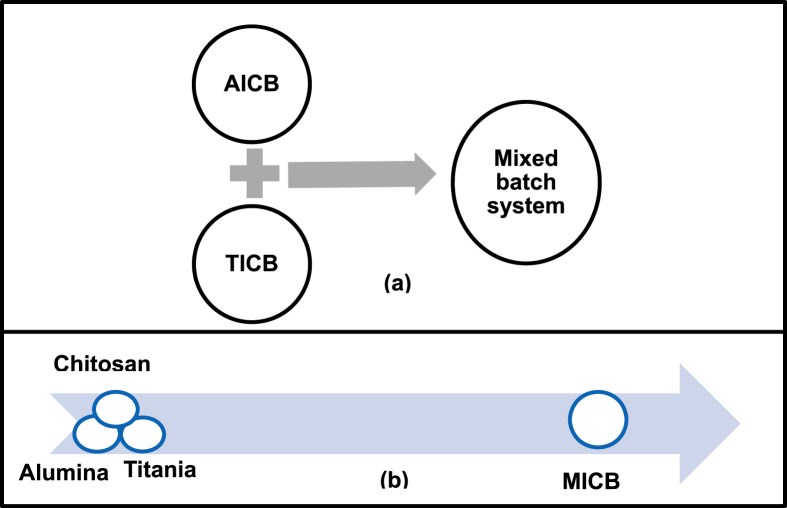
(a) Individually added alumina chitosan bead (AlCB) and titania chitosan bead (TICB) to form mixed batch system (b) mixed impregnated chitosan bead preparation (MICB) by mixing chitosan, alumina and titania.

Miller and Zimmerman [59] synthesized titanium impregnated chitosan bead (TICB) (70.2 wt% chitosan, 29.8 wt% Ti$O_{2}$) for the removal of arsenic. Experiments revealed that TICB had a greater capacity of adsorption for As (III) in the presence of UV light because of the availability of more sorption sites. It was noticed that TICB adsorbed maximum amount of arsenic within 185 hrs. Miller and Yamani *et al*, [60] developed mixed metal oxide chitosan bead by preparing the homogenous solution of chitosan in the presence of both Ti$O_{2}$ and $Al_{2}O_{3}$ and used it as an adsorbent to adsorb arsenic from aqueous media. Various types of adsorption experiments such as mixed batch and mixed bead treatment were performed in this work, as shown in Fig. 2. Kinetic study confirmed that arsenite is completely transformed into arsenate within 15 min when 1ppm arsenite solution is treated with mixed impregnated chitosan bead (MICB) ($Al_{2}O_{3}$ + Ti$O_{2}$) under UV light.

#### 2.1.2 Titania nanotubes based CNTs composites

Carbon nanotubes are being used as an adsorbent or filter for various types of pollutants i.e. organic (1, 2- dichlorobenzene and trihalomethanes, etc.) and metal ions (lead, cadmium and zinc etc.). Large number of adsorption sites available on the surface of CNTs, moreover, it has an ability of showing high heterogeneous adsorption behaviour. The adsorptive features of CNTs have been improved by coating with other metal or metal oxides i.e. manganese oxide, alumina, copper oxide and iron oxide and employed for the waste water treatment [83, 86]. Researchers have determined that CNTs-iron oxide composites showed promising results for the removal of lead (II), Cu (II), Ni (II) and cationic dyes from water. MWCNTs-copper based composites have also been utilized for the detection of optimum concentration of paracetamol and dopamine in drugs [96].

Titania nanotubes have also been used for the removal of heavy metals from water due to large surface area and it easy separation capability. However, TNTs have little adsorption capacity for metal anions due to low zero point charge, thus composites of titania nanotubes are being prepared by various methods to improve its adsorption capacity [58].

Various techniques are utilized for the production of titania nanotubes and titanium wires, have very significant role in wastewater treatment [61]. Niu *et al*, applied hydrothermal method to produce the titania nanotubes of different surface area (197–312 m^2^/g) and analyzed it for the adsorption of arsenic. Batch tests were performed to evaluate the effect of pH and results showed that TNTs removed maximum amount of arsenate in the acidic conditions while arsenite under alkaline conditions. Adsorption isotherms were used to study the influence of others co-existing ions by spiking different ionic solutions into the arsenic solution. From this experiment, it was concluded that TNTs have more affinity for As (V) and presence of other anions have less impact on the adsorption process [62].

Ti$O_{2}$-carbon nanotubes network filter were formed by filtration-steam hydrolysis method. Three sorption experiments were performed such as batch, single-pass filtration and recirculate filtration to analyze the removal efficiency of the Ti$O_{2}$-CNTs filter for arsenic. Moreover, in this study kinetic and isotherm models were used to explain the mechanism of adsorption of arsenic on this filter. The titania-CNT filter have large surface area, therefore, its adsorption capacity for both inorganic form of arsenic (As (III) and As (V)) also enhanced. The results of kinetic models indicated that adsorption of arsenite and arsenate on prepared filter followed the pseudo first order kinetic reactions and rate of reaction was 127 fold greater on Ti$O_{2}$-CNT filter than granular form of titania [63].

#### 2.1.3 Titania based MMT adsorbent

Kaolinite, montmorillonite and red mud are the types of clays that can be easily available and are used to adsorb dyes and toxic metals from water [34]. However, MMT exhibit high removal efficiency for various pollutants than other forms of clays [18]. Adsorption capacity of MMT materials have been improved by pillaring with other metal ions i.e. Al (III), Ti (IV) and iron (III) and applied for the removal of both cationic and anionic form of metals. Hydroxyl iron-MMT, hydroxyl titanium MMT and hydroxyl aluminum-MMT are used for the adsorption of cationic metals (cadmium (II), lead (II), copper (II), zinc (II) etc.) [28].

Literature study showed that, Ti$O_{2}$- pillared clay exhibited high adsorption capacity due to more porous structure, large surface area and these titania particles act as a pillars between silicate layers. Similarly, in another study, Ti$O_{2}$-monotomrolonite clay was developed via a hydrothermal method and analyzed for the photocatalytic degradation of trichloroethylene. The size of Ti$O_{2}$--MMT clay was smaller than unmodified clays which resulted in greater photocatalytic activity [64, 65].

Yuan li *et al.* synthesized Ti$O_{2}$/MMT by hydrothermal method and compared its adsorption for both form of arsenic (AsO_3_^−3^ and AsO_4_^−3^) in the presence and absence of UV light. Batch and column experiment were employed to evaluate the adsorption kinetics, it was found that UV light had significant effect on the adsorption of As (III) than As (V). In this study, the effect of pH was also studied which showed that without UV radiation, the removal of As (III) increased with an increase of pH from 3-10 and decreased with the further increase in pH. However, in the presence of ultraviolent light, pH has less effect on the adsorption of arsenite [66].

### 2.2 Titania based -metal oxide nanocomposites

#### 2.2.1 Titania based iron oxide nanocomposites

Iron oxides has been widely utilized for waste water treatment and easily separated from water due to magnetic nature of iron [26]. It was noted in different adsorption experiments that iron oxide nanoparticles also require oxidizing agents to convert As (III) into As (V). In literature reported that iron oxide nanoparticles also used for the removal of dyes i.e. methylene blue and safranin O (more toxic dyes) from natural water [87]. Therefore iron oxide doped with various metal/metal oxide materials i.e. iron-manganese, iron-copper, iron-titania, iron-aluminum, iron-carbon, Fe-CaCO_3_. GO-$Fe_{2}O_{3}$ and Fe-La (OH)_2_ etc. have been investigated for the removal of both inorganic forms of arsenic [1].

##### 2.2.1.1 *Ti*${\text{O}}_{2}$*/α-*${\text{Fe}}_{2}{\text{O}}_{3}$

**Fig. 3:**
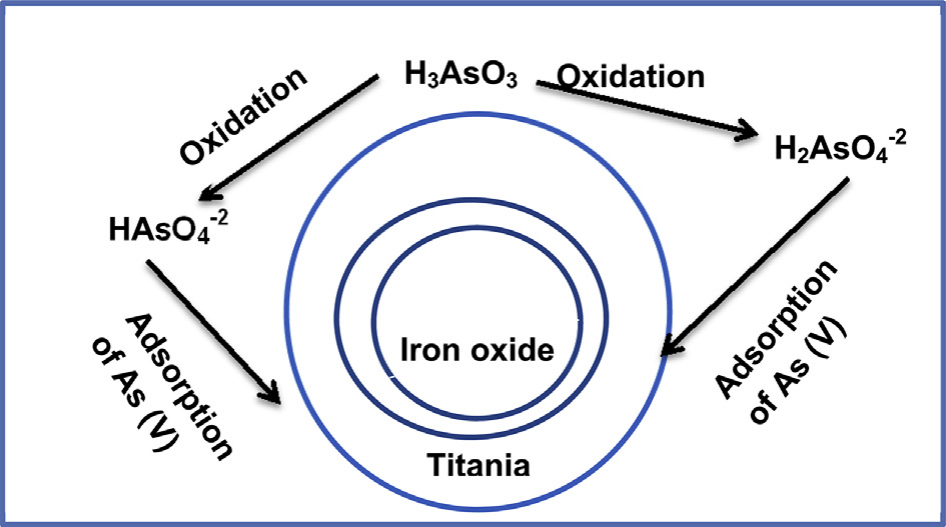
Schematic representation of adsorption of As (V) on meso- Ti$O_{2}$ /α$Fe_{2}O_{3}.\;$ H_3_AsO_3_ (As III) oxidized into As (V) i.e. HAsO_4_^−2^ and H_2_AsO_4_^−2^.

Wei t al, developed meso- Ti$O_{2}$ /α-$Fe_{2}O_{3}$, by wet impregnation method. However, the properties of both nanoparticles i.e. photocatalytic activity of Ti$O_{2}$and adsorptive feature of$\;\;\;Fe_{2}O_{3})$ remained unchanged in composite form. This composite was found efficient for the removal of arsenite. Fig. 3 represented the mechanism of adsorption of arsenic on meso- Ti$O_{2}$ /α-$Fe_{2}O_{3}$ composites. The adsorption experiment was conducted which revealed that the removal of As (III) was directly related to the pH. The effect of various concentration of iron oxide (30, 50, 70 wt %) on the titania was also studied which showed optimal efficiency for 50 wt% of$\;\;Fe_{2}O_{3}$. When experiments desorption was performed, the structure of composite remained unchanged and 94% arsenate was desorbed from adsorbent [67]. Mitch et al, synthesized Ti$O_{2}$ /α-$Fe_{2}O_{3}$ composite by easy precipitating technique and examined it for the removal of arsenic. The maximum removal of arsenic was found at pH 5. Kinetic and isotherm model showed that adsorption followed pseudo-second order kinetics [68].

Yanqi et al., used the hydrothermal treatment to fabricate Fe-TNTs and used it for the removal of arsenate. Langmuir isotherm model explained the adsorption of As (V) on Fe-TNTs, which indicated high adsorption efficiency and complete regeneration of adsorbent [69]. Kaushik Gupta et al., prepared clusters of nanostructured i.e. Iron (III)-titanium (IV) mixed binary oxide (NHITO) and tested it for the removal of arsenic. Batch experiments were conducted to examine the removal efficiency of NHITO composite for arsenite and arsenate at pH 7.0. It was found that synthesized nanostructured composite have high adsorption capacity for As (III) than As (V). This suggested that adsorption of both forms of As on the NHITO can be explained by both pseudo first and second kinetic model [70].

##### 2.2.1.2 rGO-${\text{Fe}}_{2}{\text{O}}_{3}$ /Ti${\text{O}}_{2}$ based nanocomposites

Graphene oxides have been utilized for the removal of organic and inorganic pollutants due to presence of various types of functional groups (COO^−^, OH^−^ and epoxide) on its surface [26]. The adsorption capacity of graphene oxide improved by forming its composites with other nanoparticles i.e. titania, iron oxide, manganese oxide, alumina and zinc oxide and employed for the removal of Ni^+2^, Cd^+2^, Pb^+2^, Cr^+6^ As^+3^ and As^+5^ from aqueous media [25]. GO/Au/iron oxide nanocomposites was a stable and regenerate able nanocomposite, moreover it has been proved as a good catalyst for the decomposition of nitro aromatic compounds e.g. nitrophenol compounds [90]. Silver nanoparticles with graphene oxide sheets used as a biosensor material for the detection of dietary inhibitors such as quercetin and morin [92].

Cadiam *et al* studied the prepared rGO-$Fe_{2}O_{3}$ /Ti$O_{2}$ [71] ternary composite for the removal of methylene blue under UV-light and arsenite from aqueous media. The adsorption of As (III) metal ions on this composite was described by both adsorption isotherm (Langmuir and Frendulich) and kinetics models, respectively. Field emission scanning electron microscopy (FESEM) determined the crumpled/rippled type structure of adsorbent which occurs due to the restacking of rGO. Iron oxide and titania are seen to be homogeneously dispersed on the surface of reduced graphene oxide. The process of adsorption of As (III) on rGFT was explained by Langmuir, Frendulich, Intraparticle diffusion and kinetic models. Both Freundlich and Langmuir isotherms were found to be suitable for adsorption of arsenite on these nanocomposites. The kinetic results showed that adsorption of arsenic (III) followed the pseudo second order kinetic model and more arsenic was adsorbed on adsorbent with the increase of contact time between adsorbent and adsorbate. On the basis of intra particle diffusion model (IPD) it was considered that both boundary layer and intra particle diffusion effect were significant during the adsorption of As (III). Due to the prominent features of ternary nanocomposites (rGO-$Fe_{2}O_{3}$ - Ti$O_{2}$), it can be an appropriate candidate for many applications related to waste water management.

#### 2.2.2 ${\text{Fe}}_{2}{\text{O}}_{3}$/Ti${\text{O}}_{2}$ -Si${\text{O}}_{2}$ ternary nanocomposites

Silica nanoparticles are used as an adsorbent for the removal of heavy metals, dyes and others pollutant from water. Adsorption capacity of Si$O_{2}$was not so high due to the presence of silanol groups which showed weak interactions with the metal cations. Therefore, surface of silica is chemically modified with thiol (thioether and thiourea) and amino group by surface grafting technique. These modified forms of silica have been investigated for the adsorption of copper (II), Zinc (II), Cr (III), mercury (II) and nickel (II) etc. [30].

Marzieh Sadeghil *et al.* developed a three component composite and applied for the removal of arsenic from contaminated water. $Fe_{2}O_{3}$/Ti$O_{2}$ -Si$O_{2}$modified with zinc (FTSZ) was synthesized by combination of sol-gel and wet impregnation method. Equilibrium experiment was conducted for three adsorbent i.e. iron, FTSZ, unmodified FTS & Ti$O_{2}$ -Si$O_{2}$) to compare the adsorption capacity for As and it was found that modified composite i.e. FTSZ had more affinity for As. The process of adsorption of arsenite on synthesized composite was also explained by isotherms (Langmuir and Frendulich), kinetic and intraparticle diffusion model (IPD). Theoretical and experimental $q_{e}$ values suggested that sorption of As (III) followed the second order kinetics reactions [72].

#### 2.2.3 ${\text{V}}_{2}{\text{O}}_{5}$ based Ti${\text{O}}_{2}$ nanocomposites

Vanadium nanoparticles have been modulated into different crystalline or amorphous forms without changing its nanoscale size. Silver/Vanadium-titania three component system and Vanadium- CeO_2_ composites, V_2_O_5_/TiO_2_, V_2_O_5_/ZnO, V_2_O_5_ were utilized for the photocatalytic degradation of various dyes i.e. rhodamine B (RB), coomassie brilliant blue G-250 (CBB), methylene blue and malachite green dyes from aqueous solution [73, 74, 75].

**Fig. 4:**
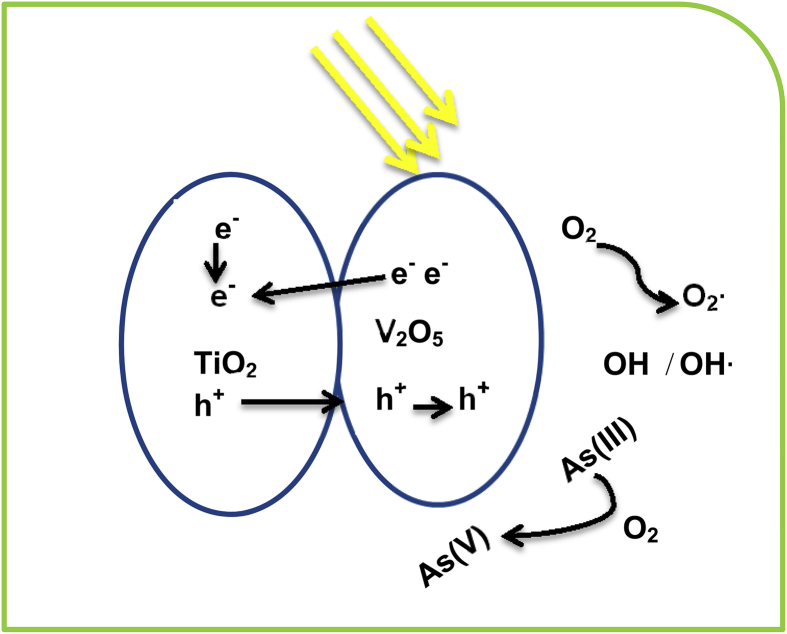
Suggested mechanism of photoxidation of arsenite on V_2_O_5_/TiO_2_ nanocomposites. Electrons and holes are generated which convert oxygen and hydroxyl into O_2_•/OH• radicals that cause the oxidation of As (III) into As (V).

Liyan Xie et al, [76] synthesized a $V_{2}O_{5}$- Ti$O_{2}$ nanocomposites in different proportions by hydrothermal and solvothermal process, and tested it for the removal of arsenic. Mechanism of removal of arsenic (III) by this composite has been shown in Fig. 4. When the visible light falls on the compound it produces various types of interactive species (hydroxyl radical, oxygen radicals etc.) which are responsible for the photocatalytic oxidation of arsenite into arsenate. ESR studies determined that interactive species such as O_2_• and h^+^ were generated when $V_{2}O_{5}$- Ti$O_{2}$ nanocomposites irradiated with visible light which are responsible for oxidation of arsenite into arsenate. In this paper, removal efficiency of core shell and solid sphere nanostructures of $V_{2}O_{5}$- Ti$O_{2}$ nanocomposites for As (III) in the presence of visible light has also been compared. From the results it was found that core shell nanostructure had high removal capacity for arsenite than solid sphere complex due to large surface area.

#### 2.2.4 Ce based TiO_2_ adsorbent

Researchers have also given attention on the use of ceria based nanoparticles for the waste water treatment due to its structural uniqueness i.e. existence in various size and shapes (flower type, nanorods, nanosphere and nanocubes). Cerium oxides nanoparticles are used for the removal of various heavy metals such as Cd (II), Pd (II), and Cr (VI) and photocatalytic degradation of dyes exist in natural water [24, 85]. Cerium based nanocomposites have also been developed for the adsorption of toxic metals for example Ce-manganese binary oxide is used for the removal of F^−^ ions and Ce-CNTs based composites are used for the removal of As (V) [30].

Zhinjian *et al*, prepared cerium-TiO_2_ hybrid adsorbent by two step reactions (hydrolysis-precipitation) and used for the removal of As (III) and As (V). Batch sorption experiments were performed to analyze the adsorbent activity, moreover, adsorption isotherms, kinetic models and effect of pH on the adsorption of arsenite and arsenate were also discussed in details. Isotherms models showed that Ce-titanium oxide hybrid adsorbent have high affinity for As (V) than arsenite (As III); maximum arsenate was adsorbed when pH was less than 7.0. X-ray photon spectroscopy confirmed that hydroxyl groups present on the surface of composite form monodentate and bidentate complexes with arsenic (As (III) and As (V)) [77]. Shubo deng *et al*, also synthesized the cerium-titania hybrid adsorbent via hydrolysis and perciptation technique followed by the addition of polyvinyl alcohol during the synthesis of composite and employed it for the removal of As (V). The purpose of addition of PVA was to increase the mechanical strength of cerium-titania hybrid adsorbent. Experiments were conducted to evaluate the effect of pH, competing anions (phosphate, sulphide, fluoride and chloride) on the adsorption of arsenate. The results indicated that adsorption of As (V) decreased in the presence of F^−^ ions as compared to chloride, phosphate and sulphide. Moreover, kinetic and adsorption isotherms results showed that adsorption of arsenate on composite followed the langmiur model and pseudo first order kinetic reactions [78].

#### 2.2.5 Zr based Ti${\text{O}}_{2}$ nanocomposites

It has been reported in various literature that zirconium based adsorbents showed maximum removal efficiency for arsenic due to high stability [1]. Zirconium oxide nanoparticles (hydrous and amorphous ZrO_2_) and Zr based composites (Zr –loaded resins and Zr- impregnated activated carbon) were also used to remove arsenic (V), however, it has an disadvantage that these composites cannot be easily separated from water [28].

Hydrous Zr$O_{2}$ has a mesoporous morphology and have high adsorption capacity for arsenate as compared to other commercially available Zr(OH)_2_ products [79]. Ivan Andjelkovic *et al,* prepared zirconium doped Ti$O_{2}$ by microwave hydrothermal method and used for the adsorption arsenic (As (III) and As (V) from aqueous media. Adsorption experiment was conducted to explain the adsorption capacity by isotherm and kinetic model. Kinetic study described that As (III) and As (V) followed pseudo first order and pseudo second order reactions, respectively. However, $R^{2}$ values suggested that adsorption mechanism of arsenite and arsenate can be better interpreted by pseudo second order. The kinetic results presented that Zr based titania composites removed maximum amount of arsenate as compared to arsenite within first 60 min. It was analyzed from isotherm models that adsorption of arsenic trioxide can be better explained by Freundlich model while As (V) adsorption followed Langmuir isotherm. When pH of solution is changed, it altered the surface charge of the composite which had a significant effect on adsorption process. Maximum arsenate adsorption was observed at pH 3 but adsorption decreased when pH was increased from 3 to 11 which may be due to increase in electrostatic repulsion between As (V) and Zr-TiO_2_ composite. However, zirconium based titania nanoadsorbent showed high affinity for As (III) in acidic conditions [80].

#### 2.2.6 Ti-loaded basic Yttrium Carbonate based nanocomposites

Literature study showed that various yttrium based compounds i.e. Yr-Mn binary oxide, Yr-iron garnate and yttrium–doped iron oxide (Fe_3_O_4_/FeOOH) have been investigated for the removal of arsenic [38]. Basic yttrium carbonate have been developed by perciptation method and used for the removal of phosphate ions [82].

Wassay et al. developed basic yttrium carbonate (BYC) by homogenous co-precipitation method and used it for the adsorption of both form of inorganic arsenic i.e. As (III) and As (V). They also studied the mechanism of adsorption, surface charge and ligand property of basic yttrium carbonate. The kinetic studies were carried out which showed that the reaction was completed within 1 hour for arsenate while arsenite adsorption took 15 hour. This suggested that the basic yttrium carbonate exhibited more affinity for As (V) than As (III) [81].

Sang ho lee *et al.* used co-perciptation method to synthesize yttrium based adsorbents i.e. yttrium based carbonate, yttrium hydroxide and titania loaded basic yttrium carbonate and employed for the removal of arsenate. Batch experiment was carried out to compare the adsorption capacity of prepared adsorbents (yttrium hydroxide, BYC and Ti-loaded BYC) for arsenate. The results indicated that Ti- loaded BYC had high adsorption capacity for arsenic (V) due to large surface area as compared to Y-OH and BYC adsorbent. It was also noticed that Ti-Loaded BYC composite adsorbed maximum amount of arsenic at pH 7.0 and performed well in the presence of competing anions (PO_4_^−2^, SiO_3_^−2^ and HCO_3_^−2^). Both Frendulich and Langmuir isotherms were used to describe the process of adsorption of As (V) on Titania loaded BYC, and the Frendulich model was found to be more favorable. It was suggested that Ti-BYC, can be applicable for long time adsorption process on the basis of its regeneration capability [82].

## 3 Conclusion

Arsenic has been becoming a global issue and causes various harmful diseases. Various conventional techniques (oxidation, coagulation, adsorption and membrane technologies etc.) are available for the remediation of arsenic contaminated water. However, among them adsorption method has been widely employed for the removal of arsenic because it is cost effective, easily operated and adsorbent can be regenerated. Researchers have found that nanoadsorbent proved to be an emerging tool for waste water treatment due to their large surface area, high adsorption capacity and high reactivity etc. Moreover, nanoadsorbent plays an efficient role in lowering the concentration of arsenic uptil permissible limit i.e.10μgL^-1^. Therefore, attention is paid on the use of nanomaterials for example CNTs/MWCNTs, Titania/TNTs, iron oxide, copper oxide nanoparticles etc. for the removal of arsenic from water. Among these nanoparticles, Ti$O_{2}$ based nanomaterials/nanocomposites showed more affinity for both form of arsenic due to high surface area to volume ratio, resistance to corrosion, non-toxicity and stability. Titania based materials become a subject for investigation of removal of arsenic from water and their high removal efficiency for arsenic encouraged researchers to develop further titania based nanocomposites. This review mainly focuses on the use of metal/metal oxide based titania nanocomposites i.e. Zr-TiO_2_, Ce-TiO_2_, Ti-BYC and iron based titania nanocomposites etc. for the treatment of arsenic contaminated water. The surface area, particle size and adsorption capacity of various titania based materials are also discussed. Adsorption of both inorganic forms of arsenic on titania based materials depend upon pH of aqueous media and well explained by Langmuir, Frendulich and IPD models. This review can be helpful in choosing the adsorbent for the removal of maximum amount of arsenic from drinking water.

## Declarations

### Author contribution statement

All authors listed have significantly contributed to the development and the writing of this article.

### Funding statement

This research did not receive any specific grant from funding agencies in the public, commercial, or not-for-profit sectors.

### Competing interest statement

The authors declare no conflict of interest.

### Additional information

No additional information is available for this paper.
